# Modelling Virus and Antibody Dynamics during Dengue Virus Infection Suggests a Role for Antibody in Virus Clearance

**DOI:** 10.1371/journal.pcbi.1004951

**Published:** 2016-05-23

**Authors:** Hannah E Clapham, Than Ha Quyen, Duong Thi Hue Kien, Ilaria Dorigatti, Cameron P Simmons, Neil M Ferguson

**Affiliations:** 1 MRC Centre for Outbreak Analysis and Modelling, DIDE, Imperial College, London, United Kingdom; 2 Oxford University Clinical Research Unit-Wellcome Trust Major Overseas Programme, Ho Chi Minh City, Vietnam; 3 Centre for Tropical Medicine, University of Oxford, Oxford, United Kingdom; 4 Department of Microbiology and Immunology, University of Melbourne, at the Peter Doherty Institute, Melbourne, Australia; Emory University, UNITED STATES

## Abstract

Dengue is an infection of increasing global importance, yet uncertainty remains regarding critical aspects of its virology, immunology and epidemiology. One unanswered question is how infection is controlled and cleared during a dengue infection. Antibody is thought to play a role, but little past work has examined the kinetics of both virus and antibody during natural infections. We present data on multiple virus and antibody titres measurements recorded sequentially during infection from 53 Vietnamese dengue patients. We fit mechanistic mathematical models of the dynamics of viral replication and the host immune response to these data. These models fit the data well. The model with antibody removing virus fits the data best, but with a role suggested for ADCC or other infected cell clearance mechanisms. Our analysis therefore shows that the observed viral and antibody kinetics are consistent with antibody playing a key role in controlling viral replication. This work gives quantitative insight into the relationship between antibody levels and the efficiency of viral clearance. It will inform the future development of mechanistic models of how vaccines and antivirals might modify the course of natural dengue infection.

## Author Summary

Dengue is a globally important viral disease. Despite this, there is still much unknown about the immunology, virology and epidemiology of dengue. As for all viral infections, the interaction between virus and immune response is a complex one. Using data collected from patients, we model how the virus replicates in an infected individual and how the human antibody response acts to control that replication. We show that the timing and magnitude of the growth and decline of virus and antibody levels in dengue-infected patients are consistent with antibody playing a key role in controlling infection. Our results are of use in the evaluation of potential antiviral drugs and vaccines.

## Introduction

In contrast to malaria, dengue is a vector-borne infection with a growing geographical range, which is therefore responsible for an increasing burden of disease [1]. Much remains to be understood about the epidemiology and pathogenesis of infection, notably how infection with one serotype modifies viral replication and disease in a later infection with a different serotype. Multiple studies have examined the role of antibody in enhancing infection [2], antigenic sin in T or B cells [2, 3] and protection afforded against infection or disease [4]. However, only a limited amount of past work has examined how the kinetics of the antibody response interact with the dynamics of viral replication within the infected patient, and investigated the causes of viral clearance during infection. Previous viral dynamic modelling work for dengue has fit mechanistic models of various immune responses to viral titres [5, 6]. Here we extend this work to fit to both viral and antibody titres during infection.

One previous study [7] analysed a small number of serial antibody measurements from primary dengue infections to examine whether antibody titres, along with NS1 measurements, could be used as an alternative diagnostic method for detecting infection. The study showed that IgM antibodies were detectable in 43% of cases on day 3 of symptoms, though in some individuals they were detectable from day 1 and were detectable in 100% of individuals by day 8. Some individuals also had detectable IgG antibodies by day 8. Though generally only two measurements were available per patient, the study highlighted high levels of heterogeneity between patients in antibody responses.

These results echo what was seen in an older study [8] which showed that in primary infection IgM antibody developed more quickly and to higher levels than IgG, but that the reverse was true in secondary infection. This work led to the use of the ratio of IgG vs. IgM titres to classify primary and secondary infection. IgM was also noted to become detectable at around the same time point as virus became undetectable, but since the main focus of the work was the use of antibody titre measurements as a diagnostic tool, mechanistic explanations of antibody and virus dynamics were not considered. Zompi and colleagues [9] considered the kinetics of antibody and B cell populations during acute secondary DENV3 infection in Nicaragua. Early in infection they found that the majority of antibody was cross- reactive with more antibody directed towards DENV2 than DENV3. Most recently, a study of Mexican patients compared (at a single time-point) viral titres in patients with or without detectable IgM [10]. Lower virus titres were observed in individuals with detectable IgM.

There are two mechanisms by which dengue infection can be controlled: limiting the rate of production of new virus particles (by blocking virus entering the cell or preventing the cell from releasing virus) or increasing the clearance of infected cells or virus (neutralisation or opsonisation and clearance). Antibody can play a role in the clearance of virus through neutralisation [11] and in the clearance of infected cells through antibody dependent cell cytotoxicity (ADCC) [12]. In this paper, we explore whether sequential antibody and virus measurements from a closely observed set of Vietnamese dengue patients are temporally and mechanistically consistent with either or both of these mechanisms for antibody action.

## Results

**Fig 1:**
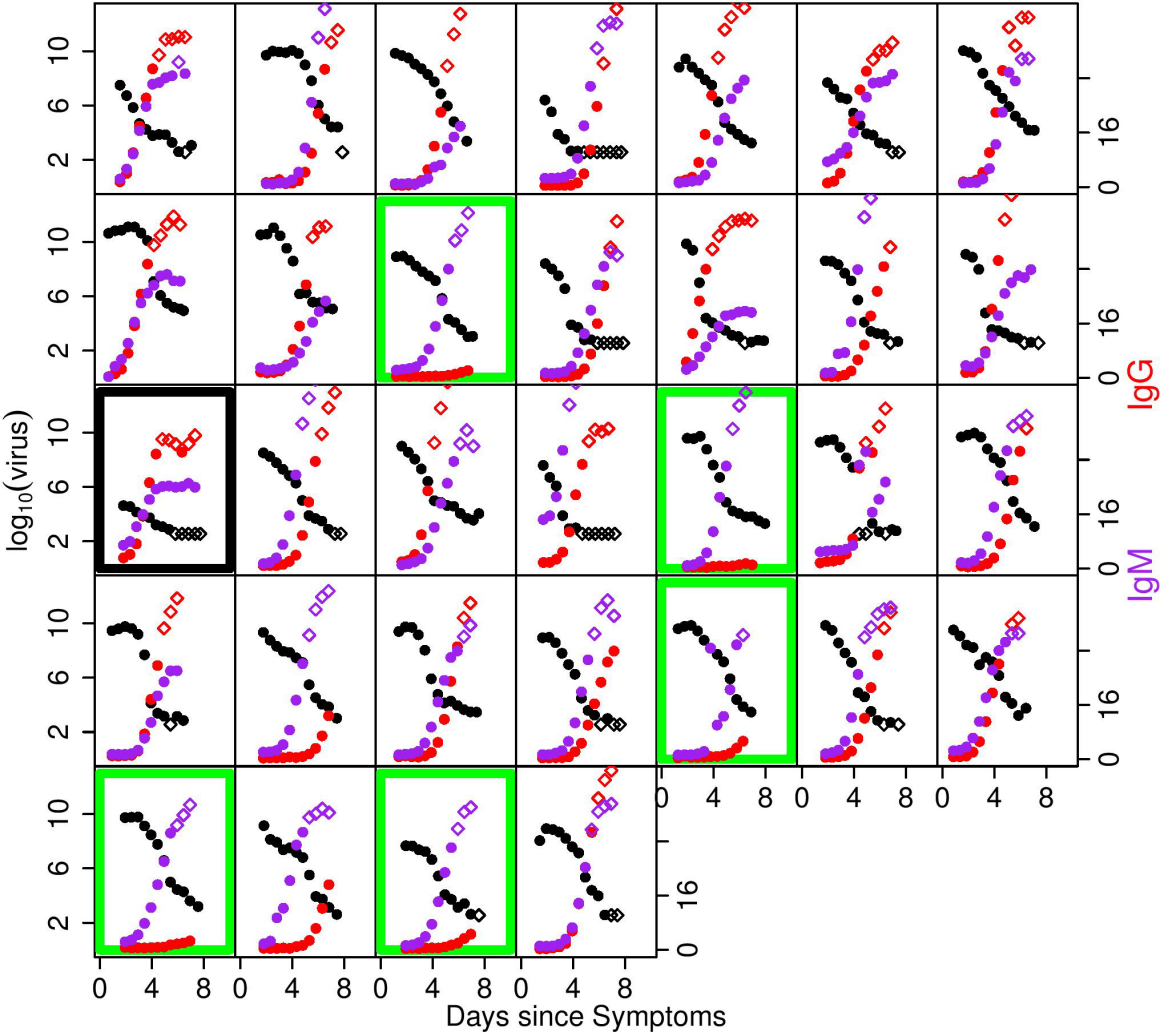
Measured RNA and antibody titres over time for DENV1-infected subjects. The left hand axis shows log_10_ RNA copies/ml of plasma in black. The right hand axis shows IgG titres in red and IgM titres in purple (both on a linear scale). Each box represents the viral and antibody measurements of a different individual. Unfilled marker symbols show measurements below the assay limit of detection for virus and above the upper limit of reliable (linear) quantification for IgG and IgM [13]. Subjects classified as primary infections are outlined in green. Patient 15, who shows the lowest RNA titres overall, is outlined in black.

**Fig 2:**
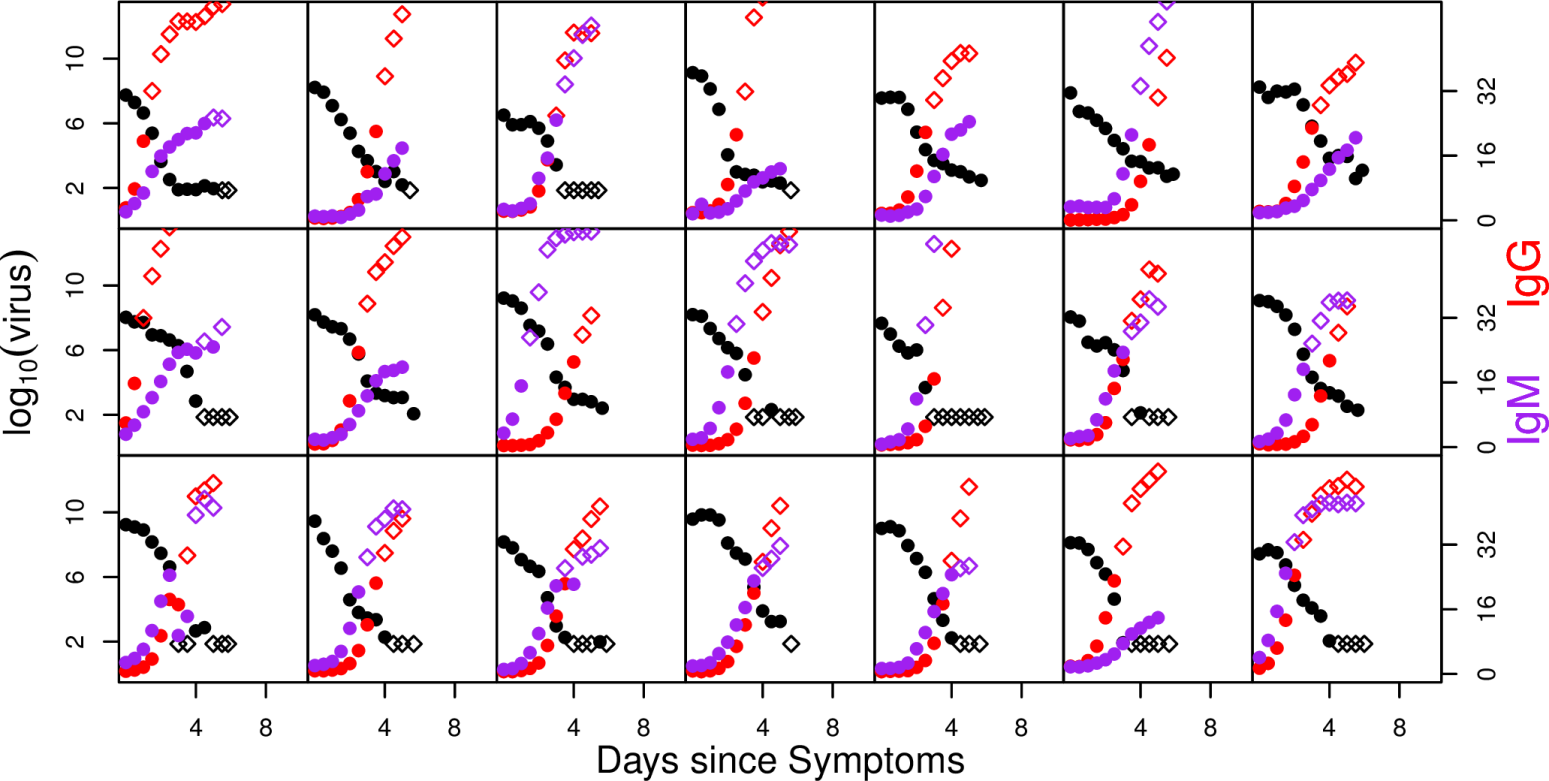
As Fig 1 but for DENV2-infected subjects. The left hand axis shows log_10_ viral copies/ml of plasma in black. The right hand axis shows IgG titres in red and IgM titres in purple (both on a linear scale). Each box represents the viral and antibody measurements of a different individual. Unfilled marker symbols show measurements below the assay limit of detection for virus and above the upper limit of reliable (linear) quantification for IgG and IgM [13]. All subjects were classified as secondary infections.

Virus and antibody titres were measured throughout DENV1 and 2 infections (Figs 1 and 2). A summary of characteristics of the dataset is given in Table 1. The levels of IgG titres in patients with primary infection were too low during infection for IgG to play a role in viral clearance. We therefore fitted the IgG data only to data from patients with secondary infections and the IgM titres to both primary and secondary infections. Since measurements only started after patients sought healthcare (and therefore after symptoms had started), data are only typically available from around the time of peak RNA titres. A peak in RNA titre (defined as an observed increase in titre relative to the first measurement, followed by a decline) was observed in 12 out of the 32 DENV1 patients and 7 of 21 DENV2 patients. Subject 15 (marked in black in Fig 1) was an outlier in having very low peak RNA titres and therefore we excluded this patient from the model fitting.

**Table 1:** Summary of data characteristics.

	DENV1	DENV2
**No. of patients**	32	21
**No. of measurements per person virus, IgG, IgM (mean, s.d.)**	11.4, 0.5	11.4, 0.5
**Day symptoms at first measurement (mean, median, s.d.)**	1.6, 1.7, 0.35	1.5, 1.6, 0.30
**% Secondary**	84	100
**RNA decrease rate log**_**10**_ **per day (mean, median, s.d.)**	-1.2, -1.3, 0.70	-1.5, -1.4, 0.40
**RNA titre at start of decrease log**_**10**_ **(mean, median, s.d.)**	9.0, 9.3, 1.2	8.2, 8.1, 0.9
**Day of RNA decrease start (symptoms) (mean, median, s.d.)**	4.2, 4.3, 1.4	3.9, 3.7, 1.5
**IgM at start of decrease (mean, median, s.d.)**	3.8, 2.4, 3.7	4.1, 2.3, 4.9
**IgG at start of decrease (mean, median, s.d.)**	1.7, 0.9, 2.8	4.5, 1.6, 11.6

**Fig 3:**
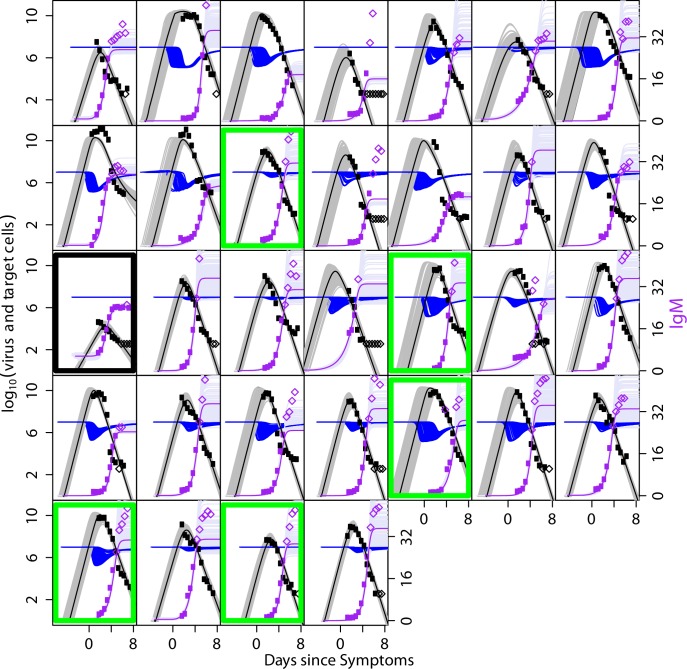
Fit of the model to virus and IgM measurements for DENV1-infected subjects for the model variant in which antibody removed virus. Each plot shows an individual patient. Virus data is shown in black and IgM data in purple. Parameters *z*_*0*_, *η*_*1*,_ *η*_*2*_, *IP* and *SF* fitted as patient specific, other parameters fitted per group. Mauve, grey and blue curves show 100 samples from the posterior distributions of antibody, virus and target cell trajectories, respectively. Median fits are shown as bold lines (purple shows IgM, black shows virus). Parameter estimates are shown in Table 2. Subjects classified as primary infections highlighted with green outline.

**Fig 4:**
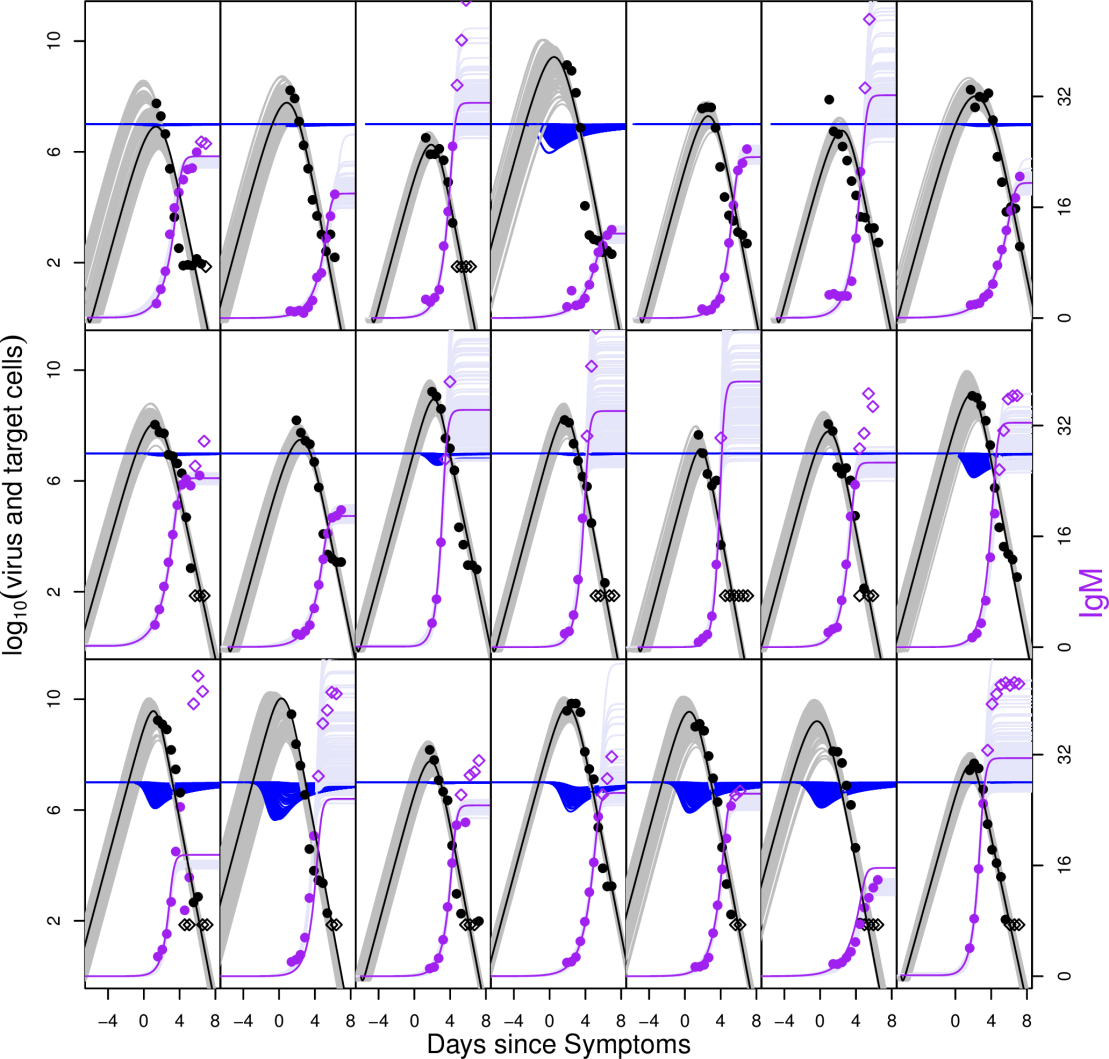
As Fig 3 but for DENV2-infected subjects. Fit of the model to virus and IgM measurements for DENV1-infected subjects for the model variant in which antibody removed virus. Each plot shows an individual patient. Virus data is shown in black and IgM data in purple. Parameters *z*_*0*_, *η*_*1*,_ *η*_*2*_, *IP* and *SF* fitted as patient specific, other parameters fitted per group. Mauve, grey and blue curves show 100 samples from the posterior distributions of antibody, virus and target cell trajectories, respectively. Median fits are shown as bold lines (purple shows IgM, black shows virus). Parameter estimates are shown in Table 2. All subjects were classified as secondary infections.

**Fig 5:**
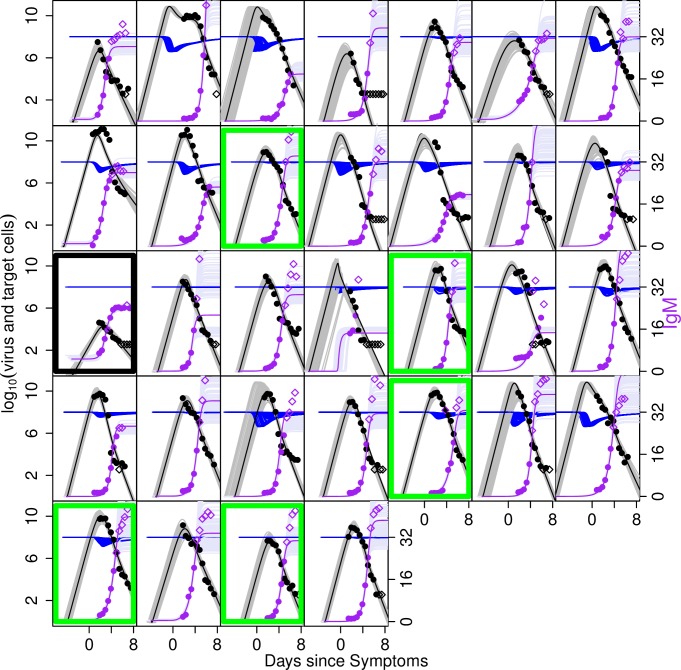
Fit of the model to virus and IgM measurements for DENV1-infected subjects for the ADCC model variant. Each plot shows an individual patient. Virus data is shown in black and IgM data in purple. Parameters *z*_*0*_, *η*_*1*,_ *η*_*2*_, *IP* and *SF* fitted as patient specific, other parameters fitted per group. Mauve, grey and blue curves show 100 samples from the posterior distributions of antibody, virus and target cell trajectories, respectively. Median fits are shown as bold lines (purple shows IgM, black shows virus). Parameter estimates are shown in Table 3. Subjects classified as primary infections highlighted with green outline.

**Fig 6:**
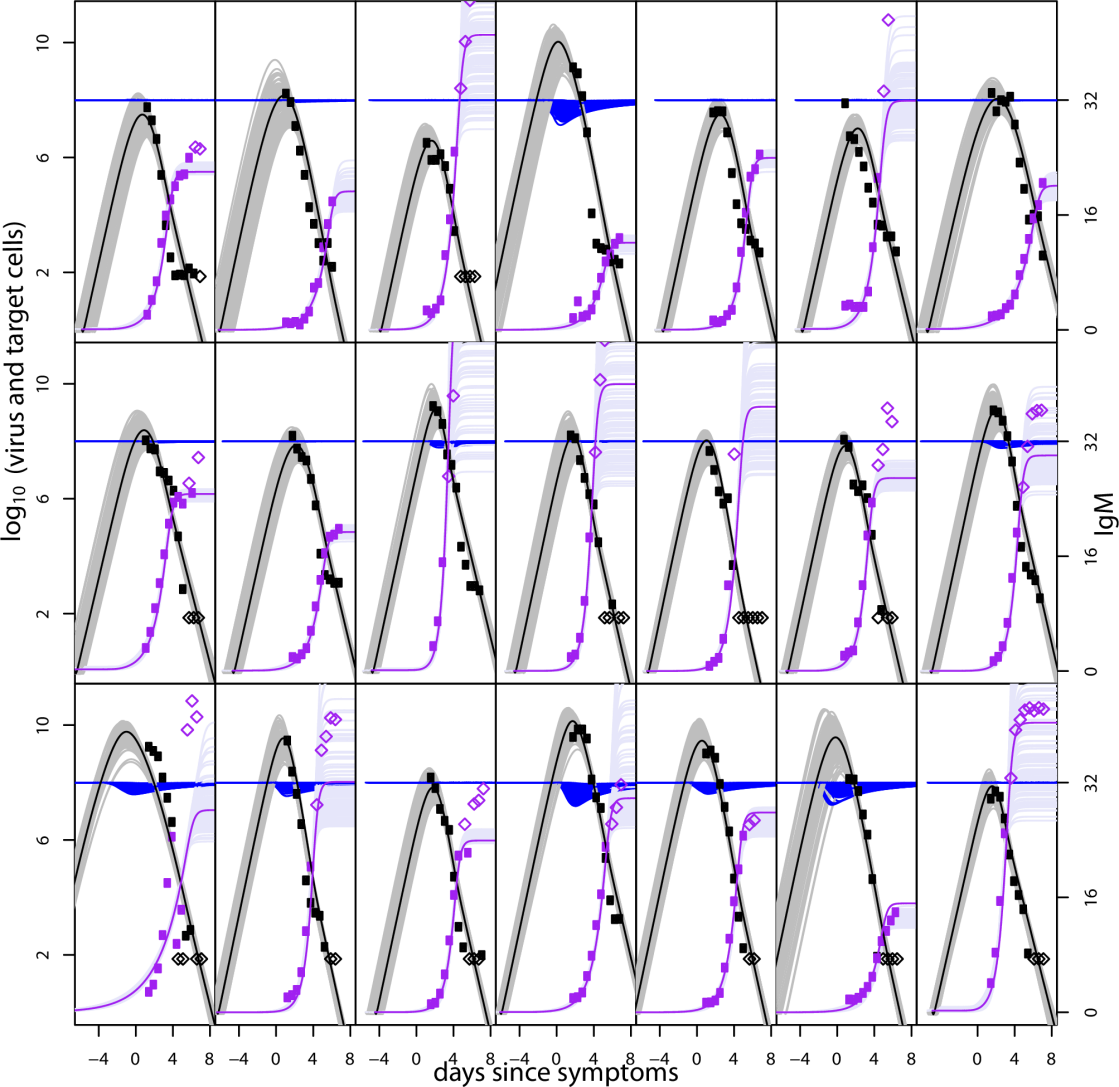
As Fig 5 but for DENV2-infected subjects. Fit of the model to virus and IgM measurements for DENV1-infected subjects for the ADCC model variant. Each plot shows an individual patient. Virus data is shown in black and IgM data in purple. Parameters *z*_*0*_, *η*_*1*,_ *η*_*2*_, *IP* and *SF* fitted as patient specific, other parameters fitted per group. Mauve, grey and blue curves show 100 samples from the posterior distributions of antibody, virus and target cell trajectories, respectively. Median fits are shown as bold lines (purple shows IgM, black shows virus). Parameter estimates are shown in Table 3. All subjects were classified as secondary infections.

We explored models of virus replication and immune control with two extreme cases for the action of antibody: direct neutralisation of free virus, and killing of infected cells (e.g. via ADCC). We found that either assumption was able to fit the data well, pointing towards a dominant role for antibody in shaping DENV RNAemia dynamics, in particular IgM. Though both models fit qualitatively well, the fit of the virus neutralisation model was statistically significantly better (judged by the log likelihood difference) than the ADCC model (Tables 2 and 3). This model fit better for 24 out of 31 individuals. We also see in comparing the fit of the antibody neutralization model to the virus and IgM antibody titres (Figs 3 and 4) with the model fit of the ADCC model (Figs 5 and 6), that the first model captures the magnitude and timing of the early viral titres better than the second. Parameter estimates for both model variants are given in Tables 2 and 3.The model fits for both models to the virus and IgG titres are shown in the Figs A-D in S1 Text with the parameters in Tables A-B in S1 Text.

**Table 2:** Parameter estimates for the model with virus neutralisation, in which antibody removes virus (Model 2). The model was separately fitted to IgM data for DENV1 and DENV2 infections. For immune response parameters and the incubation period which are patient specific parameters, median (IQR) and min and max are reported. For the group-level parameter *δ*, the median and (95% CI) are reported. Parameters z_0_, *η*_*1*,_ *η*_*2*,_ *IP* and *SF* were fitted as patient specific and others as common to all patients in the fitted group.

Serotype	Parameter	Parameter Estimates
**DENV1**	*z*_*0*_	0.121 [0.0401, 0.351] {1.70x10^-3^, 1.24}
	*η*_*1*_	1.19 [1.03, 1.38] {0.496, 2.13}
	*IP*	5.51 [4.68, 6.66] {3.88, 8.29}
	*SF*	0.177 [0.0987, 0.293] {0.017, 1.41}
	*η*_*2*_	2.41x10^5^ [7.63x10^4^, 2.28x10^6^] {1.40, 1.11x10^9^}
	*δ*	3.30 (3.07, 3.48)
	*Log-likelihood*	-539
**DENV2**	*z*_*0*_	0.177 [0.0712, 0.471] {9.54x10^-3^, 0.941}
	*η*_*1*_	1.04 [0.815, 1.22] {0.562, 1.99}
	*IP*	5.57 [4.57, 6.44] {3.54, 7.86}
	*SF*	0.151 [0.0616, 0.192] {0.0346, 0.748}
	*η*_*2*_	1.62x10^4^ [4.42x10^3^, 1.66x10^5^] {41.6, 1.22x10^7^}
	*δ*	3.54 (3.38, 3.67)
	*Log-likelihood*	-647

**Table 3:** Parameter estimates for the model with ADCC, in which antibody removes infected cells (Model 1). The model was separately fitted to IgM data for DENV1 and DENV2 infections. For patient specific parameters (i.e. the immune response parameters and the incubation period, IP), median [IQR] and {minimum, maximum} estimates across subjects are reported. For parameters assumed to be the same for all infections with the same serotype (*κ*), the median posterior estimate and 95% CI are reported. Parameters z_0_, *η*_*1*,_ *η*_*2*,_ *IP* and *SF* were fitted as patient-specific and others as common to all patients in the fitted group.

Serotype	Parameter	Parameter Estimates
**DENV1**	*z*_*0*_	0.0319 [0.0118, 0.169] {6.79x10^-4^, 0.658}
	*η*_*1*_	1.15 [0.932, 1.380] {0.489, 1.83}
	*IP*	6.26 [5.25, 7.21] {4.09, 8.55}
	*SF*	0.411 [0.22, 0.621] {0.0408, 3.65}
	*η*_*2*_	15.1 [3.29, 2.62x10^2^] {4.31x10^-4^, 2.7x10^5^}
	*κ*	4.01 (3.92, 4.10)
	*Log-likelihood*	-675
**DENV2**	*z*_*0*_	0.0418 [0.0242, 0.10] {0.00101, 0.455}
	*η*_*1*_	0.987 [0.803, 1.20] {0.479, 1.83}
	*IP*	5.95 [4.98, 6.56] {4.09, 9.10}
	*SF*	0.224 [0.151, 0.362] {0.0423, 0.714}
	*η*_*2*_	0.625 [0.0806, 8.93] {4.54x10^-4^, 2.75x10^3^}
	*κ*	3.99 (3.90, 4.14)
	*Log-likelihood*	-685

The scaling factor, *SF*, relating ELISA measured antibody levels to actual effective antibody titres, was fitted independently for each patient; this is equivalent to assuming that a specific density of antibody has differing effectiveness in clearing virus or infected cell clearance across subjects. However, the estimated differences between individuals in the value of *SF* were not large, and it is possible to fit the data reasonably (though less well, with more predicted target cell limitation) assuming this parameter takes the same value for all individuals (see Figs E and F in S1 Text).

## Discussion

In this paper, we used dynamical modelling to show that the measured titres of antibody and virus throughout dengue infection are consistent with antibody playing a dominant role in shaping virus dynamics. Antibody kinetics as measured by IgG and IgM ELISA were able to explain infection dynamics and clearance in secondary dengue cases, while only IgM kinetics were able to for primary cases. That only IgM can explain the clearance in primary cases points towards a clear role for IgM in RNA clearance. The strength of this modelling approach is that we can take into account the feedback processes between viral kinetics and the immune response; the immune response is stimulated by the virus and then acts to control viral replication.

We found that the viral and antibody data are consistent with models in which antibody acts on either the virus or infected cells. This is consistent with previous modelling work that suggested that models of target cell limitation was not able to explain viral dynamics [6]. The fit was better for the model which assumed antibody directly neutralises free virus. However the estimated infected cell lifespan was a third of a day for this model variant, a low value compared with other viral infections (e.g. HIV [14]). Such a short lifespan might suggest an additional important role for dengue infection lysing cells, ADCC or T cells in clearing infected cells later in infection (i.e. from the peak of RNA titre on) or for other immune actions still to be understood; unfortunately we do not have data on RNA or antibody titres prior to the onset of symptoms, or on measures of T cell activation.

The small but significant differences in estimates of the *SF* parameter (effectively antibody efficacy) between individuals seen in the best fit model could reflect limitations of the ELISA assay, which captures all anti-dengue antibody. This crude measure of all anti-dengue antibody will most likely include multiple different levels of responses (with different epitope-specific affinities) to each serotype. The efficacy of this response will depend on the previous infecting serotype, and how long ago this infection occurred, so will likely vary between individuals. Further work with serotype specific neutralizing titres or epitope specific measures will be of interest here.

We find that even relatively low-levels of antibody (measured by ELISA) were able to begin to control infection, possibly suggesting that the immune response substantially overshoots (in terms of antibody levels attained) compared with the minimum response required for control. Our ability to quantify the relationship between antibody and RNA titres is limited by the fact that ELISA assays give results on a linear scale, while RNA titre measurements (quantified via PCR) have a dynamic range of 5 or 6 orders of magnitude, given their measurement errors are on a logarithmic scale. Our analysis suggests antibodies start to control dengue replication at concentrations below the lower limit of quantification of the ELISA assay. Use of antibody dilution assays would therefore provide better resolution of the detailed relationship between virus and antibody kinetics and would therefore allow us to explore more rigorously whether antibody trends are consistent with antibody playing the dominant role in viral control, or whether another aspect of the immune response (e.g. the innate response) must also be playing an important role. In addition, this ‘RNAemia’ as measured by RT-PCR is an imperfect proxy of infectious virus titre, and the relationship between titre and infectiousness may well break down in the latter stages of infection- close to defervescence. Non-infectious (e.g. because it is bound to neutralising Ab) virus will nonetheless continue to give a signal in the PCR assay. Measures of infectivity of individuals throughout and particularly in the latter stages of infection will be of use to clarify the magnitude of this effect.

Previous early work by Innis et al. [8] also considered IgG and IgM dynamics during infection. We adopted the criterion proposed by that work in classifying patients without quantifiable (<10) IgG antibodies by the end of infection as primary infections and the remainder as secondary. For secondary infections, Innis et al noted IgG developing more quickly than IgM. In our data, however, we observe a range of IgG and IgM kinetics for secondary cases (Figs 1 and 2). Though IgG reaches high levels ultimately in all secondary cases, in some individuals IgG and IgM growth is concurrent, or IgM actually develops more quickly than IgG. This individual heterogeneity is consistent with observations in a recent work by Hu et al. [7]. It implies that primary/secondary classification using the IgG to IgM ratio might be highly sensitive to the timings of the measurements used.

In incorporating a single monolithic immunity variable and the one to one relationship of clearance, our model makes highly simplifying assumptions about the development and binding of the immune response to dengue virus. In reality there are probably multiple arms of the immune response contributing to the control of viral replication and a more complex binding process occurring [15]. For example, in addition to B-cell mediated responses considered here, there is evidence from mice that the innate immune response may assist in viral clearance and that T-cells may be important [16]. Our model currently predicts some role for target cell depletion in infection dynamics, which may be the result of our model fits adjusting to cope with the absence of data on other parts of the immune response. Measurements of anti-viral innate immune responses (such as nonspecific Type I interferon activation) and T cell response dynamics throughout infection, paired with virus titres, will therefore be informative in disentangling which arms of the immune response play the dominant role at which stage of pathogenesis. To understand the antibody response further, multiple antigen-specific antibody measurements (possibly coupled with measurements of the capacity of sera to neutralise/enhance) would be highly informative. It would be particularly valuable to obtain such data (from human challenge models or otherwise) from early in infection, as currently we have little data on the early growth kinetics of virus or the immune response.

An understanding of how the dynamics of virus replication and the immune response interact during infection gives insight into pathogenesis and how disease course might be modulated. In this paper, the fit of mathematical models of immune system and viral dynamics to dengue patient data, sheds light on this key relationship. We have presented the first study which quantitatively and mechanistically links measured dengue virus and antibody dynamics throughout infection. We found a mathematical model of dengue antibody playing a role in controlling infection was consistent with the RNA and antibody titres throughout dengue infection.

## Materials and Methods

### Ethics statement

The trial protocol was approved by Oxford University Tropical Research Ethical Committee and the Scientific and Ethical Committee of the Ministry of Health, Vietnam. The trial was registered at http://www.clinicaltrials.gov (NCT01096576).

### Virus and antibody titres

We use RNA titre data presented in a prior publication of a clinical trial of the drug balapavir to treat dengue infection [13]. Informed consent was obtained from the study participants as described in [13]. That study saw no differences between treatment arms, so both were combined and used here. Patients were enrolled within 48 hours of fever onset. The trial had 32 subjects with DENV1 infection and 21 with DENV2 infection. All patients had twice daily viral load measurements [13].

Antibody titre measurements were also measured throughout infection in this study, and these data are presented for the first time here. IgG and IgM antibodies were measured using an ELISA assay [17–19] with quantitation via measurement of optical density. The ELISA assay does not measure antibody to a specific epitope, but overall binding of the antibody to virus. Measurements are thought to be linearly proportional to total binding levels below 25 optical density units, but above 25 the relationship becomes non-linear as optical density measurements saturate at high levels of binding. We excluded one patient (patient 15 in Fig 1) from the analysis due to the outlier virus and immune titres seen.

Using the antibody titre measurements, individuals could be classed as primary or secondary infections [8]. We categorised patients as primary infections if they had IgG titres less than 10 in the specimen collected at the time of patient discharge from hospital and as secondary (or later) infections otherwise. Using this algorithm, 5 of the DENV-1 cases were classified as primary and the remainder as secondary infections. All DENV-2 cases were classed as secondary infections.

### Mathematical model

We extended an existing mathematical model of dengue virus and immune dynamics within a host [5] to explore the extent to which antibody kinetics are consistent with a key role for antibody in limiting dengue infection. In this model (similar to those used for influenza [20, 21]), the target cells (*x*) and free virus (*v*) interact to infected cells (*y*), which can then go onto produce more virus. Whilst this is occurring, antibody levels (*z*) are increasing with the aim of halting infection (and in future providing protection against a subsequent infection). The following equations define the model.

$$\begin{array}{l} \frac{dx}{dt}=A-\gamma x-\beta xv\\ \frac{dy}{dt}=\beta xv-\delta y-\alpha zy\\ \frac{dv}{dt}=\omega y-\kappa v-\varepsilon zv\\ \frac{dz}{dt}=f(v,y,z) \end{array}$$ (1)

Parameters of the model and their meaning are given in Table 4. We fit two model variants representing different mechanisms of antibody action: Model 1: antibody acting to kill infected cells e.g. via antibody dependent cell cytotoxicity (ADCC), and Model 2: antibody neutralising and clearing the virus. We model antibody acting to kill infected cells by assigning ε = 0 and α > 0 and virus neutralization and clearance by assigning α = 0 and ε > 0.

**Table 4:** The parameters of the model and values if fixed. References for fixed parameter assignments are given.

Parameter	Meaning	Value/Estimated	Reference
***A***	Constant target cell production/ml/day	1.4x10^6^	
***γ***	Cell death rate/day	1/7 per day	
***β***	Infection rate of target cells per virion	Model 1: Primary 1.72x10^-10^ Secondary: 2.5 x10^-10^ Model 2: Primary: 3.83x10^-11^ Secondary: 5 x 10^−11^	
***α***	Removal rate of infected cells/day per immune cell	Model 1: 1 Model 2: 0	arbitrary
***ε***	Removal rate of virus/day per virion	Model 1: 0 Model 2: 1	arbitrary
***δ***	Infected cell death rate/day	Model 1:1/7 per day Model 2: estimated	[26]
***ω***	Production rate of virions/day per infected cell	1x10^4^	[27]
***κ***	Virion clearance rate/day	Model 1: estimated Model 2: 3.5 per day	From influenza
***η***_***1***_	Proliferation rate of immune cells/day per infected cell	Estimated	-
***η***_***2***_	Threshold parameter for the immune response proliferation	Estimated	-
***SF***	Scaling factor of immune response	Estimated	-
***z***_***0***_	Initial population size of immune effector population	Estimated	-
***v***_***0***_	Initial inoculum of virus	1/ml	arbitrary
***y***_***0***_	Initial population size of infected cells	0	Assuming virus is inoculated
***x***_***0***_	Initial population size of target cells	Model 1: 10^7^ Model 2: 10^8^	
***IP***	Incubation period	Estimated	-

Key to modelling the interaction between viral and immune system dynamics is how the different parts of the immune response proliferate in response to infection, represented in the model by the function *f* (*y*, *v*, *z*). In preliminary fitting we saw that the mass action formulation [5], *f* (*y*, *v*, *z*) *= ηyz* was unable to fit the IgG and IgM data. Hence we used a more realistic saturating function of infected cell or virus density: *f* (*y*, *v*, *z*) *=* (*η*_*1*_ *y z* / (*η*_*2*_ *+ y*)) (infected cell killing model) or *f* (*y*, *v*, *z*) *=* (*η*_*1*_ *v z* / (*η*_*2*_ *+ v*)) (virus neutralisation model), respectively. This functional form implicitly incorporates the processes of B cell maturation and antibody production, and similar forms have been used to model immune cell proliferation to viral infection in previous work [22].

We simultaneously fitted both viral titres and antibody levels. In order to assess which antibody measures best explained viral dynamics, we fitted the models separately to IgG and IgM data, fitting the same viral titre data in each case. Since model (1) allows for both target cell-limited and immune control of the virus, how it fits the data will shed light on the mechanism driving infection control. If the antibody and the virus dynamics are consistent with a model in which antibody is controlling virus, we would expect to find a minimal role for target cell limitation [23].

The models were fitted using Markov Chain Monte Carlo (MCMC) methods, implemented in R [24] and C, as described in previous work [5]. For each subject we estimate the length of the incubation period (i.e. the time from infection to symptom onset) using the reported day of symptom onset and an informative prior distribution for the incubation period. Informed by early human challenge studies [25], the prior distribution used was a left-truncated normal with mean of 5.7 days and standard deviation of 3 days. Antibody measurements were also included in the model likelihood, with the upper limit of reliable quantification modelled using the cumulative distribution function. The likelihood for a single subject is:

$$\prod_{i=1}^{n}\;\phi {\left(\log\;D_{iv}|\log(v_{i}),\sigma_{v}{}^{2}\right)}^{1-c_{iv}}\Phi {\left(\log\;LD_{v}|\log(v_{i}),\sigma_{v}{}^{2}\right)}^{c_{iv}}\phi {\left(D_{ia}|SFz_{i},\sigma_{a}{}^{2}\right)}^{1-ca_{i}}(1-\Phi {\left(LD_{a}|SFz_{i},\sigma_{a}{}^{2}\right)}^{ca_{i}})$$

Here *ϕ* and Φ are the probability density function and cumulative density function of the normal distribution, respectively. The number of observations for a single individual is denoted by *n*, *D*_iv_ is the *i*th viral titre measurement and *v*_i_ is the model prediction of viral titre at the *i*th measurement. *LD*_v_ is the limit of detection of viral titre and *σ*_v_ is the error of viral titre measurements on a log10 scale, assumed to be 1. The indicator function, *c*_iv_ is 0 if *D*_iv_ > *LD*_v_ and 1 if not. In addition, *D*_ia_ is the *i*th antibody level measurement, *z*_i_ is the model prediction of antibody level at the *i*th measurement and *SF* is a scaling factor for antibody measurements (discussed below). *LD*_a_ is the upper limit of reliably quantification of antibody levels and *σ*_a_ is the error in antibody measurements assumed to be 1. The indicator function, *c*_ai_ = 1 if the *i*th antibody level measurement is above the limit of reliable quantification (*LD*_a_), and 0 if not.

Since the optical density based measurements of antibody levels obtained via the ELISA assay do not provide a direct measurement of antibody density per ml of plasma, we introduce a fitted multiplicative scaling factor, *SF*, to transform the state variable *z*, which represents antibody densities in the model, into the same scale as the antibody level measurements.

The full likelihood is given by the product of the likelihood over all patients and we use the natural logarithm of the likelihood (log-likelihood) as a measure of goodness of fit.

We fit some model parameters as patient-specific and others as group-specific, with the groups here being defined by the infecting serotype (DENV1 or DENV2). Parameters relating to the host immune response (*z*_*0*_, *η*_*1*_, *η*_*2*_ and *SF*) were treated as patient-specific, whilst virus parameters (*β* and *κ and δ*) were assumed to be the same for all subjects. The assignment *α* = 1 or ε = 1 and does not affect model results; *α* and *z*_0_ are unable to be estimated simultaneously since the antibody level measurements available are relative, not absolute measures of antibody density. We estimated *κ* and fixed *β* and *x*_*0*_ to values from previous work; higher for secondary than primary cases, however the results are not sensitive to these values, and with different values for each of the model formulations (required for each model to reach the peak titers) [5]. Table 4 gives a complete list of model parameters and definitions.

## Supporting Information

S1 Text Description of results of fitting model variants. (PDF)

S1 Data Virus (PCR), IgG and IgM (ELISA) titers data. (XLSX)
