# Supplementary material for: Modelling Virus and Antibody Dynamics during Dengue Virus Infection Suggests a Role for Antibody in Virus Clearance
Source: PLoS Comput Biol. 2016 May 23;12(5):e1004951. doi: 10.1371/journal.pcbi.1004951 (PMC4877086; doi:10.1371/journal.pcbi.1004951)
Supplement: S1 Text — (PDF) [file pcbi.1004951.s001.pdf]

Supplementary Information

Modelling virus and antibody dynamics during dengue infection suggests a role for antibody in virus clearance

Short title: Modelling dengue virus and antibody dynamics

Hannah E. Clapham[1], Than Ha Quyen[2], Duong Thi Hue Kien[2], Ilaria Dorigatti[3], Cameron P. Simmons[4], Neil M. Ferguson[3]

1: MRC Centre for Outbreak Analysis and Modelling, DIDE, Imperial College, London, W2 1PG, Current address: JHSPH, Department of Epidemiology, 615 N Wolfe St, Baltimore, MD 21224
2: Oxford University Clinical Research Unit-Wellcome Trust Major Overseas Programme, Ho Chi Minh City, Vietnam.
3: MRC Centre for Outbreak Analysis and Modelling, DIDE, Imperial College, London, W2 1PG,
4: Oxford University Clinical Research Unit-Wellcome Trust Major Overseas Programme, Ho Chi Minh City, Vietnam; Centre for Tropical Medicine, University of Oxford, Oxford, United Kingdom; Department of Microbiology and Immunology, University of Melbourne, Melbourne, Australia.

Corresponding author: Hannah Clapham, Current address: JHSPH, 615 N Wolfe St, Baltimore, MD 21224, hclapha1@jhu.edu, hannah.e.clapham@gmail.com

Classification: Biological Sciences; Computational Biology; Immunology and Inflammation
Keywords: Dengue, Within-host modelling, Immune dynamics

Figure ~~S1~~A: Fit of the model to virus and IgG measurements for only secondary DENV1-infected subjects for the model variant in which antibody removed infected cells. Each plot shows an individual patient. Virus data is shown in black and IgG data in red. Parameters z_0, η_1, η_2, *IP* and *SF* fitted as patient specific, other parameters fitted per group. Pink, grey and blue curves show 100 samples from the posterior distributions of antibody, virus and target cell trajectories, respectively. Median fits are shown as bold lines (red shows IgG, black

shows virus). Parameter estimates are shown in Table ~~S1~~A.

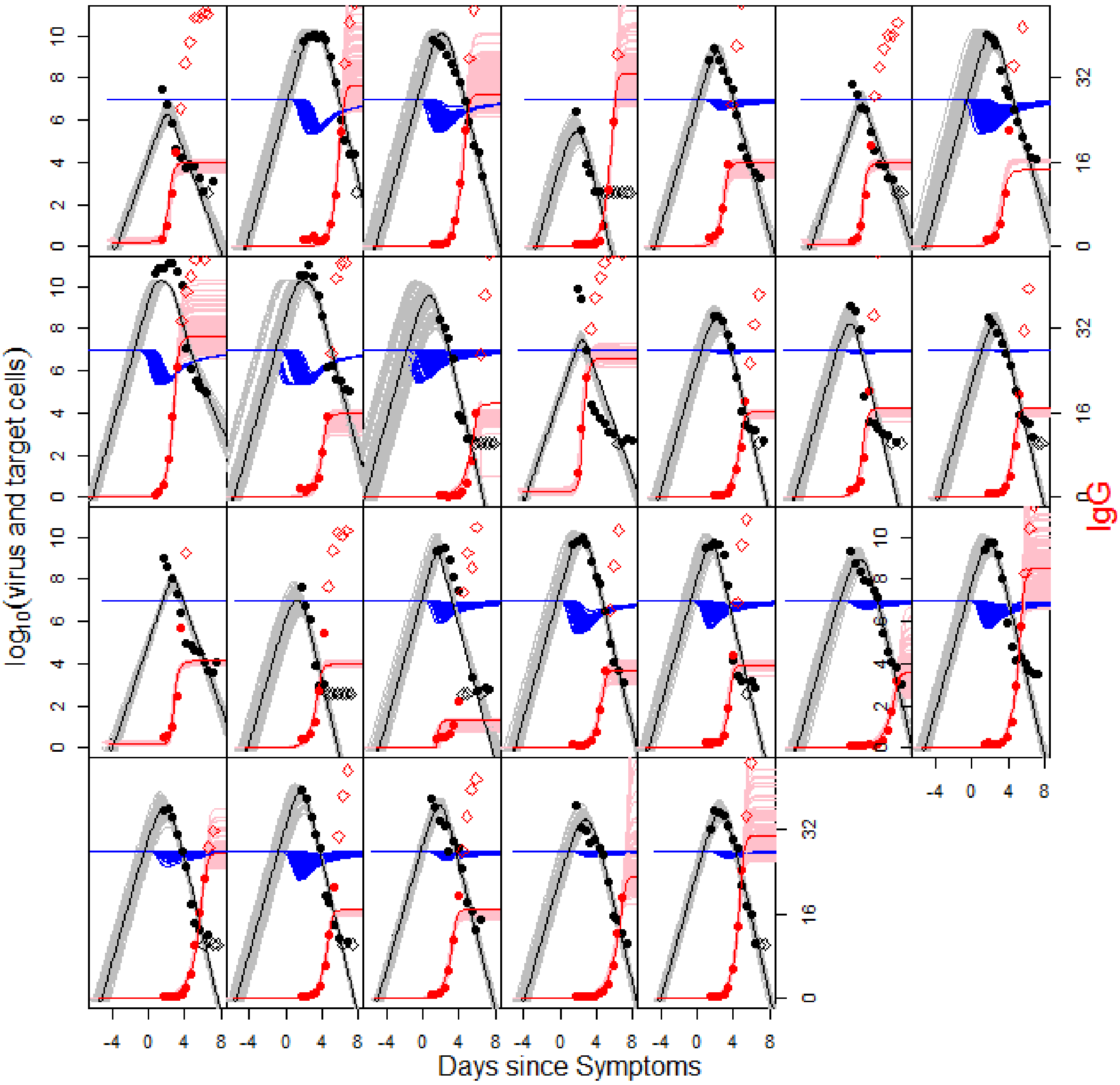

Figure ~~S2~~B: Fit of the model to virus and IgG measurements for DENV2-infected subjects for the model variant in which antibody removes infected cells. Each plot shows an individual patient. Virus data is shown in black and IgG data in red. Parameters z_0, η_1, η_2, *IP* and *SF* fitted as patient specific, other parameters fitted per group. Pink, grey and blue curves show 100 samples from the posterior distributions of antibody, virus and target cell trajectories, respectively. Median fits are shown as bold lines (red shows IgG, black shows virus). Parameter estimates are shown in Table ~~S1~~A.

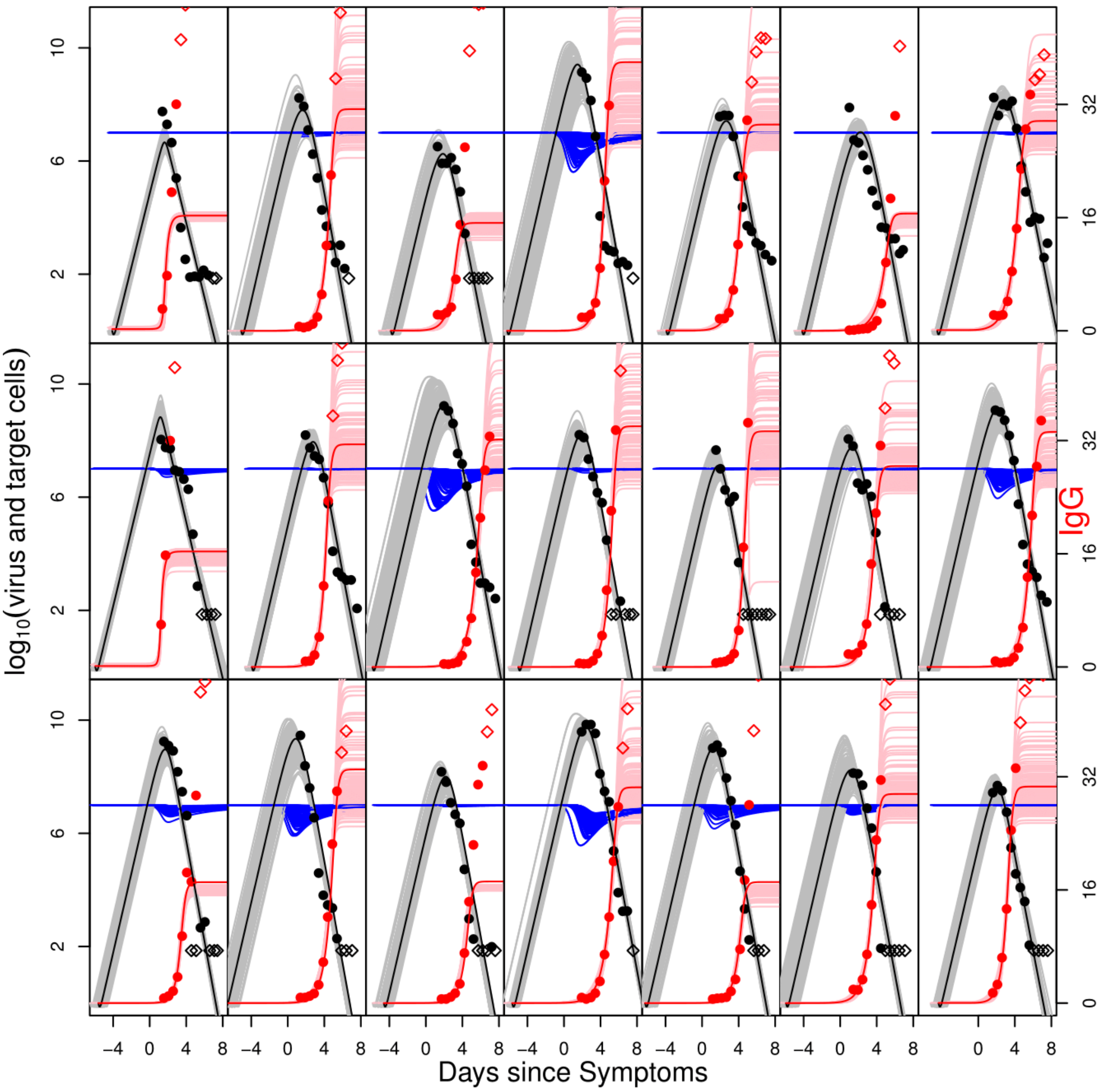

Figure ~~S3~~C**:** Fit of the model to virus and IgG measurements for only secondary DENV1-infected subjects for the model variant in which antibody removes virus. Each plot shows an individual patient. Virus data is shown in black and IgG data in red. Parameters z_0, η_1, η_2, *IP* and *SF* fitted as patient specific, other parameters fitted per group. Pink, grey and blue curves show 100 samples from the posterior distributions of antibody, virus and target cell trajectories, respectively. Median fits are shown as bold lines (red shows IgG, black shows virus). Parameter estimates are shown in Table ~~S2~~B.

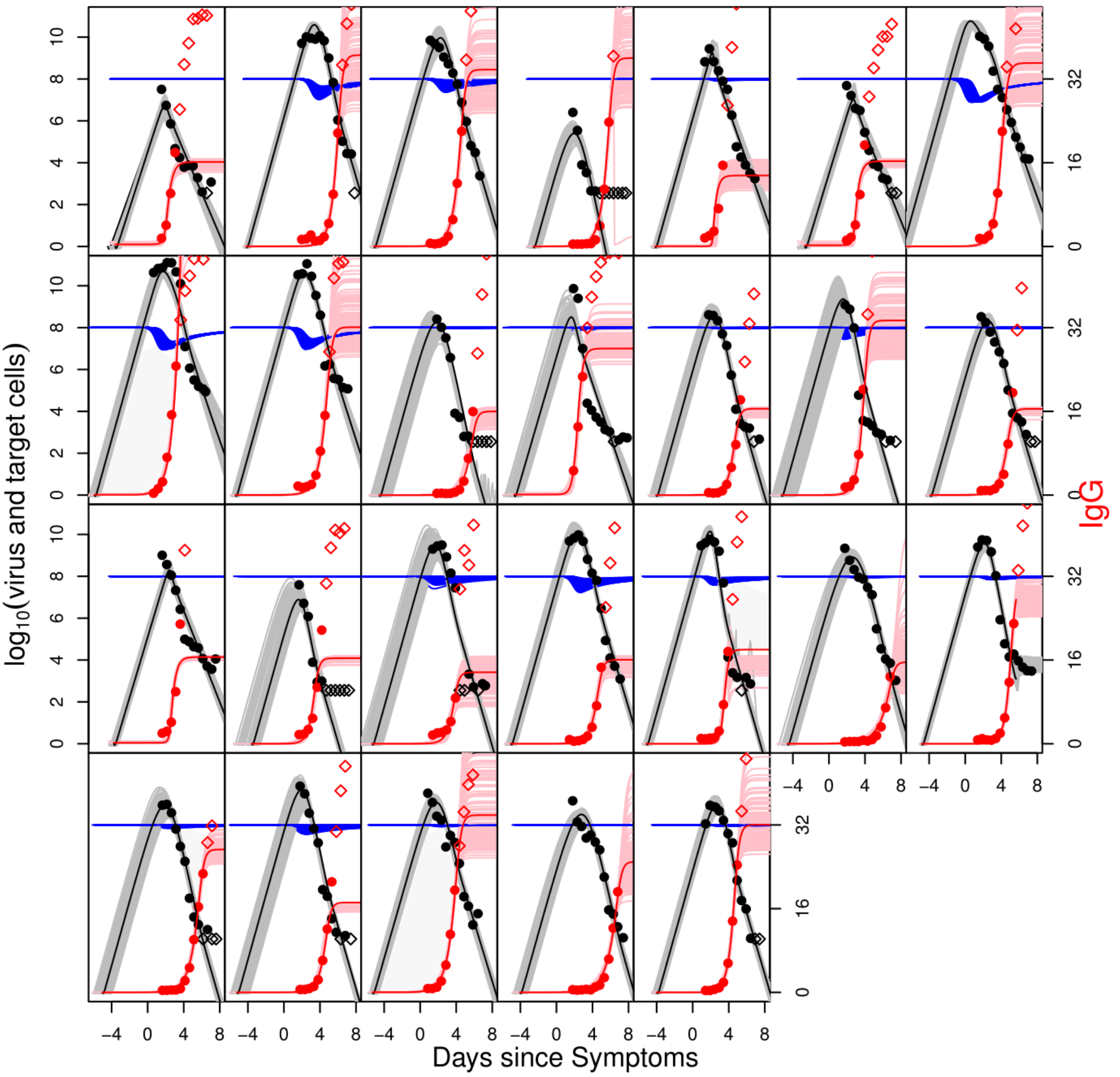

Figure S4D: Fit of the model to virus and IgG measurements for DENV2-infected subjects for the model variant in which antibody removes virus. Each plot shows an individual patient. Virus data is shown in black and IgG data in red. Parameters z_0, η_1, η_2, *IP* and *SF* fitted as patient specific, other parameters fitted per group. Pink, grey and blue curves show 100 samples from the posterior distributions of antibody, virus and target cell trajectories, respectively. Median fits are shown as bold lines (red shows IgG, black shows virus). Parameter estimates are shown in Table S2B.

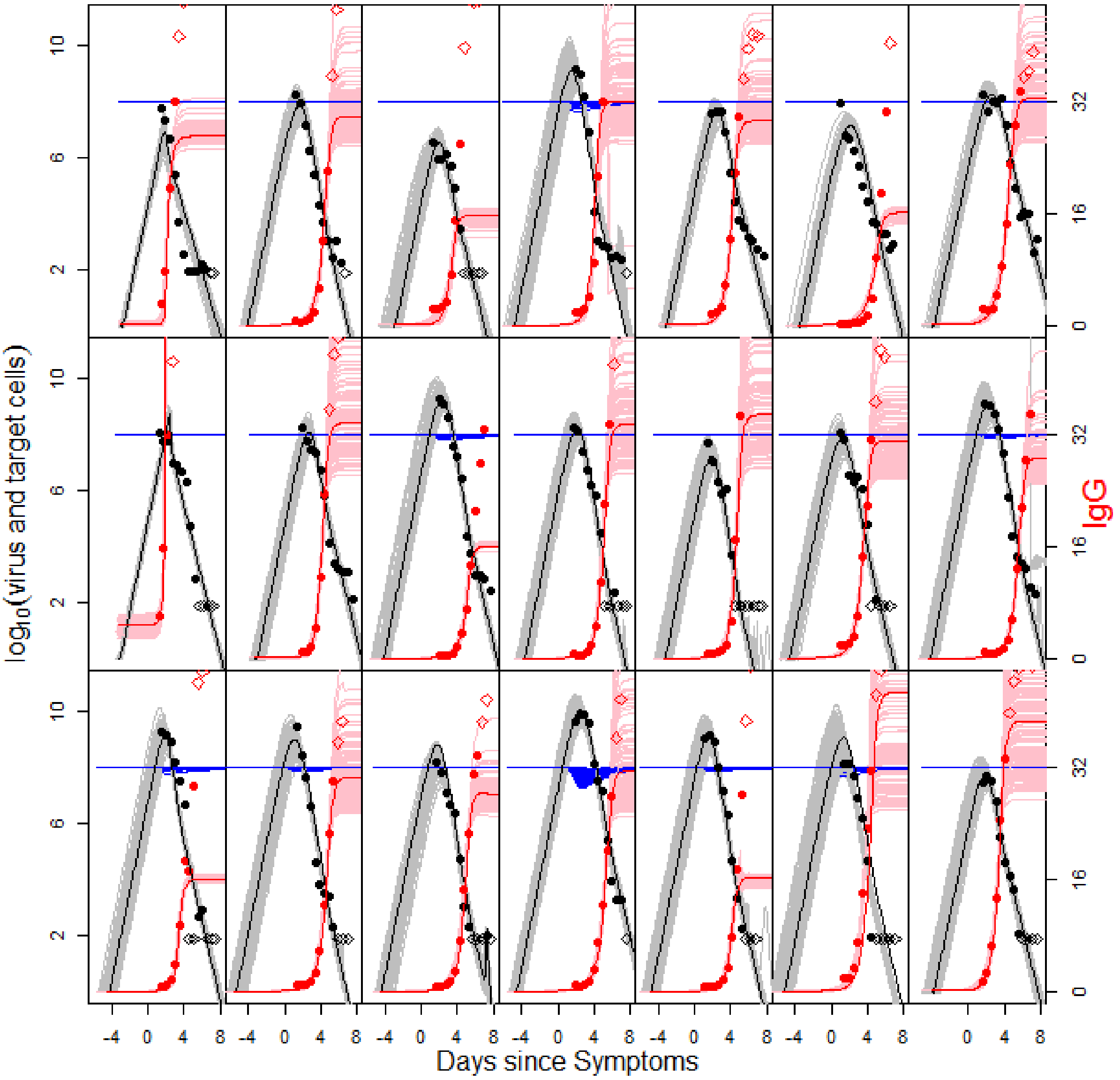

Figure ~~S5~~E: Fit of the model to virus and IgM measurements for DENV1-infected subjects for the model variant in which antibody removed virus, **but with the scaling factor parameter estimated as common to all subjects, compared to being patient specific in the main paper.** Each plot shows an individual patient. Virus data is shown in black and IgM data in purple. Parameters z_0, η_1, η_2, and *IP* fitted as patient specific, other parameters fitted per group. Mauve, grey and blue curves show 100 samples from the posterior distributions of antibody, virus and target cell trajectories, respectively. Median fits are shown as bold lines (purple shows IgM, black shows virus). Subjects classified as primary infections highlighted with green outline. **The loglikelihood of this model is -759, and the estimate of the scaling factor parameter is 0.093 and 95% CI (0.080, 0.111). This model fits the data fairly well, but not as well as the model with the patient level scaling factor (loglikelihood: -539).**

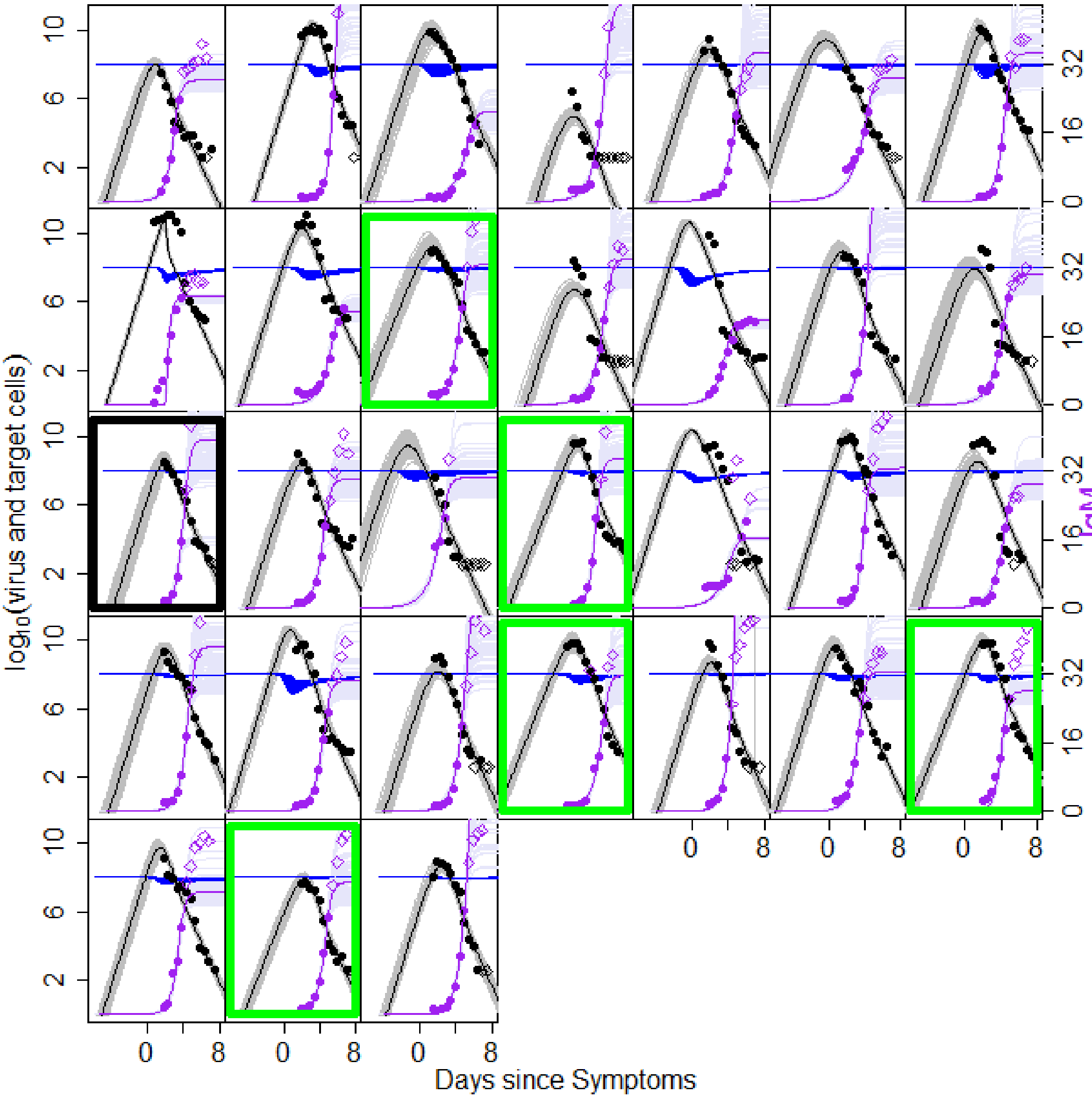

Figure ~~S6~~F: Fit of the model to virus and IgM measurements for DENV2-infected subjects for the model variant in which antibody removed virus, **but with the scaling factor parameter estimated as common to all subjects, compared to being patient specific in the main paper.** Each plot shows an individual patient. Virus data is shown in black and IgM data in purple. Parameters z_0, η_1, η_2, and *IP* fitted as patient specific, other parameters fitted per group. Mauve, grey and blue curves show 100 samples from the posterior distributions of antibody, virus and target cell trajectories, respectively. Median fits are shown as bold lines (purple shows IgM, black shows virus). **The loglikelihood of this model is -757, and the estimate of the scaling factor parameter is 0.086 (95% CI: 0.078, 0.110). This model fits the data fairly well, but not as well as the model with the patient level scaling factor (loglikelihood: -647).**

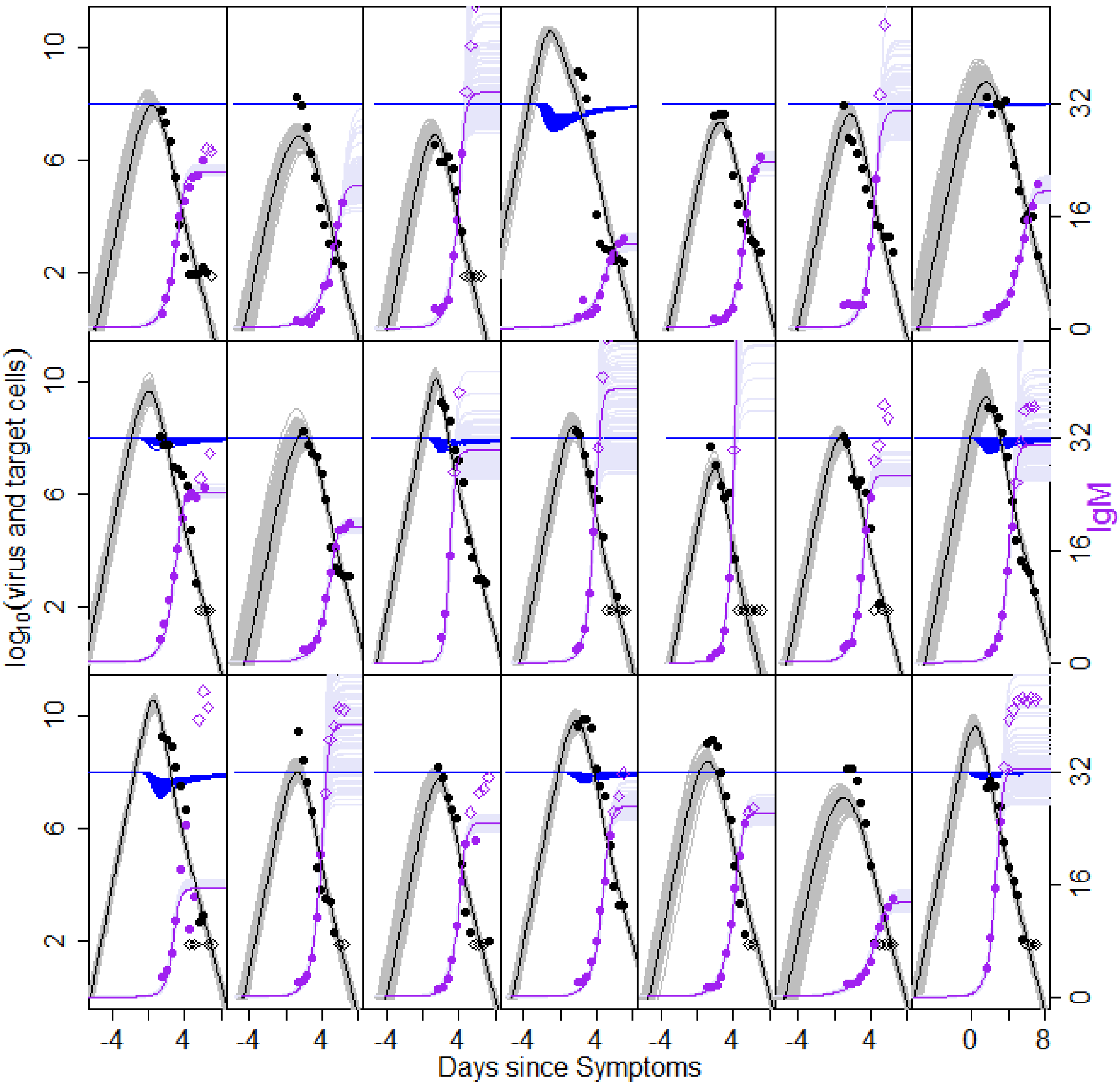

Table ~~S1~~A: Parameter estimates for the model with ADCC, in which antibody removes infected cells. The model was separately fitted to IgG data for DENV1 and DENV2 infections. For patient specific parameters (i.e. the immune response parameters and the incubation period, IP), median [IQR] and {minimum, maximum} estimates across subjects are reported. For parameters assumed to be the same for all infections with the same serotype (β and κ), the median posterior estimate and 95% CI are reported. Parameters z_0, η_1, η_2, *IP* and *SF* were fitted as patient-specific and others as common to all patients in the fitted group.

Serotype	Parameter	Parameter Estimates
**DENV1**	z_0	0.0134 [3.27×10^{-3}, 0.0341] {7.42×10^{-4}, 1.11}
	η_1	1.54 [1.40, 1.84] {1.00, 8.64×10^{200}}
	IP	4.86 [4.30, 5.49] {2.93, 6.74}
	κ	4.33 (4.23, 4.42)
	SF	0.139 [0.0507, 0.647] {0.0135, 3.69}
	η_2	3.53 [1.39e-01 ,1.38e+04] {1.83e-07, 7.58e+203}
	Log likelihood	-720
**DENV2**	z_0	6.46×10^{-3} [1.43×10^{-3}, 0.0692] {2.54×10^{-4}, 0.367}
	η_1	1.38 [1.29, 1.70] {1.08, 2.11×10^{15}}
	IP	5.11 [4.29, 5.85] {3.83, 6.89}
	κ	4.17 (4.08, 4.26)
	SF	0.083 [0.041, 0.244] {0.0160, 0.648}
	η_2	0.196 [7.67×10^{-3}, 16.6] {4.68×10^{-4}, 9.69×10^{17}}
	Log likelihood	-482

Table ~~S2~~B: Parameter estimates for the model with virus neutralisation, in which antibody removes virus. The model was separately fitted to IgG data for DENV1 and DENV2 infections. For immune response parameters and the incubation period which are patient specific parameters, median (IQR) and min and max are reported. For the group-level parameters, β and κ the median and (95% CI) are reported. Parameters z_0, η_1, η_2, *IP* and *SF* were fitted as patient specific and others as common to all patients in the fitted group.

Serotype	Parameter	Parameter Estimates
**DENV1**	z_0	$4.72\text{x}10^{-2}$ [$1.67\text{x}10^{-2}$, $8.88\text{x}10^{-2}$]<br>{$1.59\ \text{x}10^{-4}$, 0.996}
	η_1	1.59 [1.37, 2.00]<br>{0.916, $7.75\text{x}10^{233}$}
	IP	4.64 [3.99, 5.18]<br>{2.55, 7.06}
	δ	3.50 (3.49, 3.52)
	SF	$7.17\text{x}10^{-2}$ [$2.96\text{x}10^{-2}$, 0.297]<br>{7.23x10-3, 4.48}
	η_2	$8.19\text{x}10^{5}$ [$1.78\text{x}10^{4}$, $9.81\text{x}10^{6}$]<br>{0.0384, $1.13\text{x}10^{230}$}
	Log likelihood	-557
**DENV2**	z_0	$3.45\text{x}10^{-2}$ [$1.24\text{x}10^{-2}$, 0.192]<br>{$4.96\text{x}10^{-6}$, 0.556}
	η_1	1.62 [1.40, 1.82]<br>{1.07, $2.45\text{x}10^{5}$}
	IP	4.28 [3.45, 4.75]<br>{2.96, 5.52}
	δ	3.28 (3.19, 3.28)
	SF	$4.39\ \text{x}10^{-2}$ [$1.65\text{x}10^{-2}$, 0.140]<br>{$9.15\text{x}10^{-3}$, $9.56\text{x}10^{5}$}
	η_2	$2.37\text{x}10^{4}$ [$1.34\text{x}10^{3}$, $2.10\text{x}10^{5}$]<br>{$1.17\text{x}10^{2}$, $1.45\text{x}10^{12}$}
	Log likelihood	-414
